# Single‐Nucleus Atlas of Cell‐Type Specific Genetic Regulation in the Human Brain

**DOI:** 10.1002/alz70855_097916

**Published:** 2025-12-23

**Authors:** Gabriel E Hoffman

**Affiliations:** ^1^ Icahn School of Medicine at Mount Sinai, New York, NY, USA

## Abstract

**Background:**

Genetic risk variants for common diseases are predominantly located in non‐coding regulatory regions and modulate gene expression. Although bulk tissue studies have elucidated shared mechanisms of regulatory and disease‐associated genetics, the cellular specificity of these mechanisms remains largely unexplored.

**Method:**

This study presents a comprehensive single‐nucleus multi‐ancestry atlas of genetic regulation of gene expression in the human prefrontal cortex, comprising 5.6 million nuclei from 1,384 donors of diverse ancestries. Through multi‐resolution analyses spanning eight major cell classes and 27 subclasses, we identify genetic regulation for 14,258 genes, with 857 showing cell type‐specific regulatory effects at the class level and 981 at the subclass level.

**Result:**

Colocalization of genetic variants associated with gene regulation and disease traits uncovers novel cell type‐specific genes implicated in Alzheimer's disease, which were not detectable in bulk tissue analyses. Analysis of dynamic genetic regulation at the single nucleus level identifies 2,073 genes with regulatory effects that vary across developmental trajectories, inferred from a broad age range of donors. We also uncover 1,655 genes with trans‐regulatory effects, revealing distal regulation of gene expression.

**Conclusion:**

This high‐resolution atlas provides unprecedented insight into the cell type‐specific regulatory architecture of the human brain, and offers novel mechanistic targets for understanding the genetic basis of Alzheimer's disease.

